# Alveolar recruitment maneuver in refractory hypoxemia and lobar atelectasis after cardiac surgery: A case report

**DOI:** 10.1186/1749-8090-7-58

**Published:** 2012-06-22

**Authors:** Marcus Vinicius Herbst-Rodrigues, Vitor Oliveira Carvalho, Ludhmila Hajjar Abrahao, Emilia Nozawa, Maria Ignez Zanetti Feltrim, Filomena Regina Barbosa Gomes-Galas

**Affiliations:** 1 Physiotherapy Department, Heart Institute (InCor), University of Sao Paulo Medical School, Sao Paulo, Brazil; 2 Serviço de Cirurgia Cardíaca Pediátrica do Instituto do Coração do Hospital das Clínicas da Faculdade de medicina da USP (InCor-HCFMUSP), Sao Paulo, Brazil; 3 Anesthesiology Division, Heart Institute (InCor), University of Sao Paulo Medical School, Sao Paulo, Brazil; 4 Av. Dr. Enéas de Carvalho Aguiar, 44. Serviço de Fisioterapia, Bloco 1, 2° Andar, InCor, São Paulo CEP: 05403-900, Brazil

## Abstract

**Objective:**

This case report describes an unusual presentation of right upper lobe atelectasis associated with refractory hypoxemia to conventional alveolar recruitment maneuvers in a patient soon after coronary artery bypass grafting surgery.

**Method:**

Case-report.

**Results:**

The alveolar recruitment with PEEP = 40cmH_2_O improved the patient’s atelectasis and hypoxemia.

**Conclusion:**

In the present report, the unusual alveolar recruitment maneuver with PEEP 40cmH_2_O showed to be safe and efficient to reverse refractory hypoxemia and uncommon atelectasis in a patient after cardiac surgery.

## Background

The most common cardiovascular postoperative complications in cardiac surgery are related to extracorporeal circulation and its inflammatory reaction. It is well known that extracorporeal circulation affects the lungs causing alveolar edema, hypoxemia and atelectasis, which can delay extubation [1].

One strategy used to reverse the hypoxemia caused by alveolar collapse is alveolar recruitment maneuvers (ARM) [2]. Most of the times, alveolar recruitment maneuvers are performed increasing positive end-expiratory pressure on the respiratory system (PEEP) to 20 or 30 cmH_2_O with a satisfactory result in the correction of hypoxemia in patients soon after cardiac surgery.

This case report aims to describe an unusual presentation of right upper lobe atelectasis associated with refractory hypoxemia to conventional alveolar recruitment maneuvers in a patient soon after coronary artery bypass grafting surgery.

## Case report

**Figure 1:**
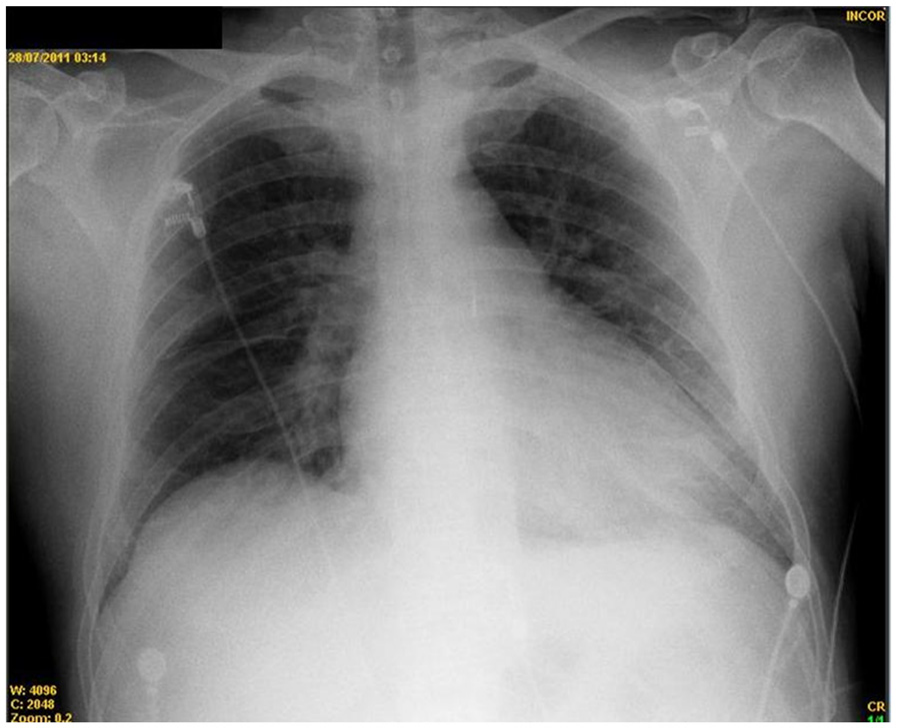
Pre-operative X-ray showing normal lung fields.

A 53-year-old, diabetic, male patient with ischemic cardiomyopathy (left ventricle ejection fraction of 20 %) in use of intra-aortic balloon (1:1) with normal X-Ray (Figure 1) was submitted to coronary artery bypass grafting surgery (extracorporeal circulation time of 106 minutes, clamping time of 61 minutes, the bypass include: left internal thoracic artery to left anterior descending artery, greater saphenous vein to diagonal branch and greater saphenous vein to obtuse marginal branch) in our hospital in July 2011.

The patient (ASA III) received midazolam orally (10 mg) administered one hour before surgery. The anesthetic technique is standardized at our institution. We used propofol for induction of anesthesia in combination with fentanyl hydrochloride. The neuromuscular blocking agent used was cisatracurium. Soon after surgery, the sedated (Ramsay 3–4) patient was referred to the intensive care unit under mechanical ventilation (VIASYS™ AVEA™, volume-controlled ventilation, Synchronized Intermittent Mandatory Ventilation + Pressure Support Ventilation, VCV-SIMV + PSV, of 8 ml\kg, total respiratory rate of 12 breaths per minute, PEEP of 5cmH_2_O, FiO_2_ of 0.6 and PSV of 10cmH_2_O) in infusion of dobutamine 16 mcg/Kg/min, noradrenaline 0.3 mcg/Kg/min, Levosimendan 5 μg/kg/min and intra-aortic balloon of 1:2. Sequential blood gas analysis and biochemical tests before and after surgery are showed in Table 1.

**Table 1:** Patient’s data before and after alveolar recruitment maneuver

**Arterial blood gas test**	**Beginning surgery (OR)**	**Ending surgery (OR)**	**Arriving ICU**	**After first ARM (30cmH**_**2**_**O)**	**After second ARM(40cmH**_**2**_**O)**	**After extubation**
**pH**	7.41	7.38	7.32	7.39	7.40	7.38
**P**_**a**_**CO**_**2**_**(mmHg)**	37	32.3	37.2	32.7	31.3	32.9
**P**_**a**_**O**_**2**_**(mmHg)**	88.8	154	69.2	75.9	155	126
**P**_**a**_**O**_**2**_**/ FiO**_**2**_	148	250	115	126	258	315
**SatO**_**2**_**(%)**	95.9	98.6	90.5	94.1	98.2	97.9
**HCO3 (mEq/L)**	23.1	18.8	18.4	19.3	18.8	19.1
**BE**	−0.7	−5.0	−6.6	−4.4	−4.7	−4.7
**Hematocrit (%)**	37	30	36	37	32	31
**Hemoglobin (g/dL)**	11.9	9.6	11.6	12.1	10.3	10.1
**Sodium (mEq/L)**	137	131	133	135	137	137
**Potassium (mEq/L)**	4.0	4.7	3.8	4.2	4.0	3.8
**Lactate (mg/dL)**	10	10	17	15	12	11
**Respiratory rate (**breaths per min**)**	12	12	12	18	15	14
**Tidal Volume (mL)**	640	640	640	640	640	----

OR: Operation room; ICU: Intensive care unit; ARM: Alveolar recruitment maneuver; P_a_CO_2_: partial pressure of carbon dioxide in arterial blood; P_a_O_2_: partial pressure of oxygen in arterial blood ;SatO_2_: Oxygen saturation in arterial blood ;BE: base excess;.

**Figure 2:**
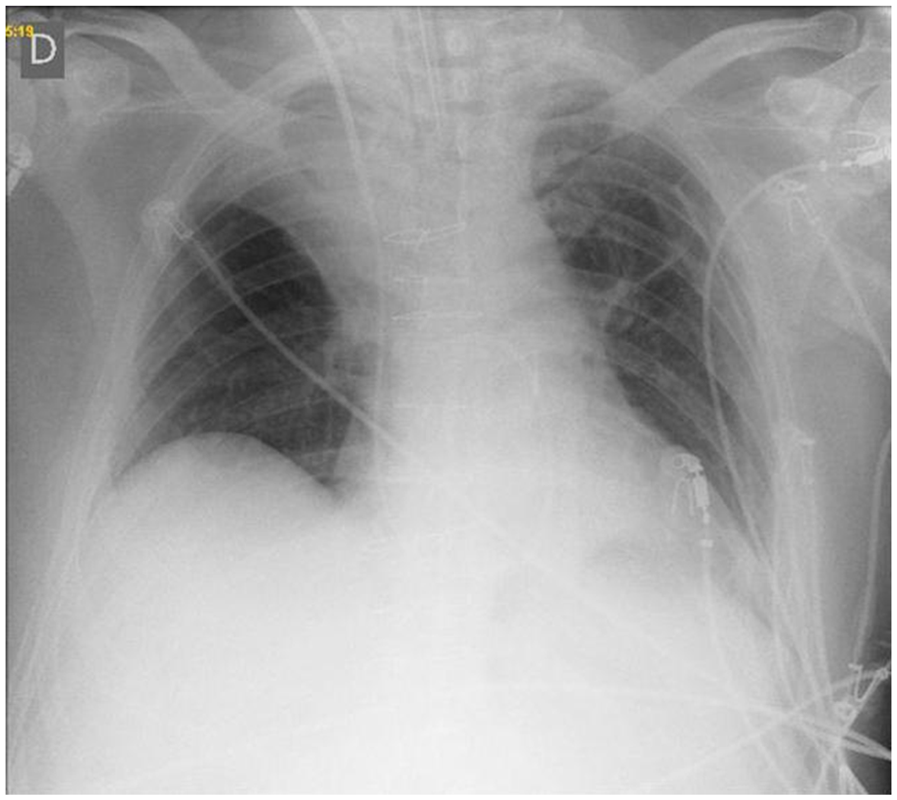
Unusual atelectasis in upper right lobe after cardiac surgery.

According to the first blood gas analysis in the intensive care unit (PaO_2_\FiO_2_ < 200), the patient was included in the hospital protocol for alveolar recruitment maneuver [3]. In addition, patient’s X-Ray showed an unusual atelectasis in upper right lobe (Figure 2). The alveolar recruitment maneuver was performed in CPAP mode of 30cmH2O and delta pressure PSV of 10cmH_2_O, maintained for 30 seconds, followed by three consecutive maneuvers with 30 seconds of interval. This methodology is standardized in our hospital routine. Alveolar recruitment maneuver was performed increasing PEEP to 30 cmH_2_O for 30 seconds (3 times) with an interval of 30 seconds between maneuvers.

**Figure 3:**
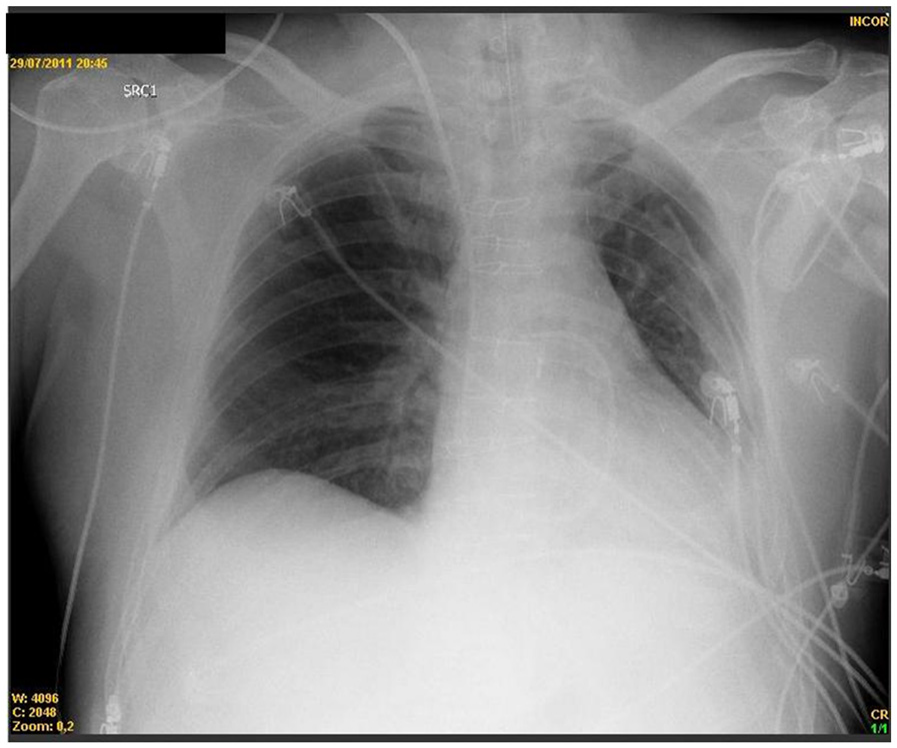
**Total resolution of atelectasis after alveolar recruitment maneuver with 40cmH**_**2**_**O.**

**Figure 4:**
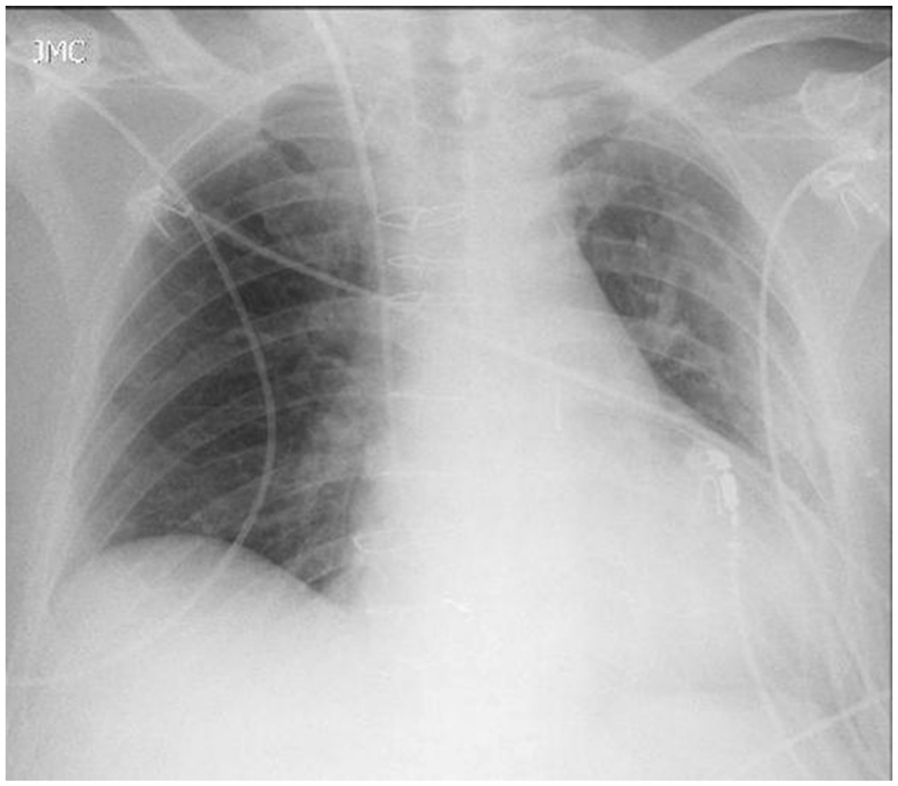
X-Ray after extubation remained without atelectasis.

After the alveolar recruitment with PEEP of 30cmH_2_O (peak inspiratory pressure of 40cmH_2_O and PSV 10cmH_2_0), the patient did not show great improvement in oxygenation (Table 1). This time, our staff decided to perform a more aggressive alveolar recruitment maneuver increasing PEEP to 40cmH_2_O (peak inspiratory pressure of 50cmH_2_O). The patient did not now show any hemodynamic alterations or pulmonary complications like pneumothorax during these alveolar recruitment maneuvers. After the alveolar recruitment with 40cmH_2_O, the patient showed a great improvement in oxygenation and total resolution of atelectasis, as demonstrated in X-Ray (Figure 3). One hour after the recruitment maneuver, the mechanical ventilation was discontinued with success. Blood gas analysis, biochemical tests and X-Ray after extubation remained in normal values (Table 1 and Figure 4).

## Discussion

In this report, the studied patient showed great improvement in oxygenation and total resolution of atelectasis after unconventional alveolar recruitment maneuver in post-operatory cardiac surgery (Table 1).

In the past decade, the PEEP therapy was used with some restrictions due to hemodynamic side effects in these kind of patients. After the study by van den Berg et al [4] this paradigm was changed. Airways pressures up to 20 cmH_2_O showed minimal effects on cardiac output, primarily because of an in-phase-associated pressurization of the abdominal compartment associated with compression of the liver and squeezing of the lungs [4].

The use of alveolar recruitment maneuver with PEEP between 20 and 30 cmH_2_O has been shown to be efficient in reversing hypoxemia after cardiac surgery [5]. Despite this, its effect in atelectasis is poorly described. In our case report, the use of 30cmH_2_O of PEEP was not enough to reverse hypoxemia and atelectasis after cardiac surgery. This way, we proposed the use of alveolar recruitment maneveur with PEEP 40 cmH_2_O (peak inspiratory pressure of 50 cmH_2_O, PSV = 10cmH_2_O) with total reversion of hypoxemia and atelectasis.

A lobar atelectasis, especially of an upper lobe, is very different from diffuse microatelecatasis, which has been extensively studied in patients with acute lung injury. Most, if not all, studies using ARM to treat "atelectasis" were actually done in patients with diffuse microatelectasis, or alveolar collapse, which is seen in dependent regions of the lungs (usually postero-inferior regions in supine patients). This difference should be the core of the discussion. The Authors should provide their interpretation as to why an ARM would be useful in this case.

In this patient, the cause of the atelectasis is unknown, but some hypotheses can be raised. The patient did not use double-lumen endotraqueal tube and selective intubation did not happen either. One possibility is mucus plugging and the other possibility is the hypoventilation during the transport of the patient, since they are manually ventilated in our service.

In unconventional situations like refractory hypoxemia associated with atelectasis, the use of alveolar recruitment maneuvers should be considered since the patient is hemodynamically stable and normovolemic. Despite this, new studies must be performed to ensure the safety and systematic indications of high levels of PEEP during alveolar recruitment maneuvers in patients after cardiac surgery.

## Conclusion

In the present report, the unusual alveolar recruitment maneuver with PEEP 40cmH_2_O showed to be safe and efficient to reverse refractory hypoxemia and uncommon atelectasis in a patient after cardiac surgery.

## Consent

Consent was obtained from the patient for publication of this case-report and any accompanying images.

## Competing interests

The authors declare that they have no competing interests.

## Authors’ contribution

MVHR and VOC collected the data, MVHR and VOC wrote the manuscript and all authors discussed the case and approved the final version of the manuscript. All authors read and approved the final manuscript.
